# Early Years Practitioners' and Public Health Consultants' Perspectives on the Use of Interactive Electronic Devices in Young Children: A Qualitative Study

**DOI:** 10.1111/cch.70022

**Published:** 2025-01-12

**Authors:** Liane B. Azevedo, Megan Downes, Sara Eastburn, Jane Covell, Paul Bissell

**Affiliations:** ^1^ College of Health, Wellbeing and Life Sciences Sheffield Hallam University Sheffield UK; ^2^ School of Human and Health Sciences University of Huddersfield Huddersfield UK; ^3^ Department of Allied Health Professions, School of Human and Health Sciences University of Huddersfield Huddersfield UK; ^4^ Kirklees Council Huddersfield UK; ^5^ University of Chester Chester UK

**Keywords:** mobile phones, nursery, tablets, thematic analysis, young children

## Abstract

**Background:**

Interactive electronic devices (IEDs) are ubiquitous in young children's lives. However, research on their impact on learning and development is still limited. The aim of this study was to understand the perspectives of early years practitioners (EYPs) and public health consultants (PHCs) on the use of IEDs in children aged 3–5.

**Methods:**

Using purposive sampling techniques, we recruited four EYPs and two PHCs from children's nurseries and a government organisation in the northwest of England. Semi‐structured interviews were used to collect data, which were audio‐recorded, transcribed verbatim and anonymised. Data were analysed using reflective thematic analysis.

**Results:**

EYPs and PHCs noted that although IEDs could negatively impact child development and behaviour, they could also aid in learning. EYPs expressed concerns about the impact of parents' own IED habits on children's communication and social skills. On the other hand, PHCs stressed that substituting outdoor play with the use of these devices could affect children's social and physical skills and reduce physical activity levels, which are crucial for development. Finally, both EYPs and PHCs agreed that there was a need to improve parents' and EYP's knowledge and to develop interactive interventions to promote an understanding of how IEDs should be used with young children.

**Conclusion:**

EYPs and PHCs acknowledge the potential advantages of using IEDs as a teaching tool for children. However, they have concerns about the long‐term effects on communication, social and physical skills and how children are impacted by their parents' use of these devices. To support policy statements, future research should offer further evidence of the benefits and harms of IED use.

**Summary**

- The views of early years practitioners (EYPs) and public health policymakers on children's use of interactive electronic devices (IEDs) have not yet been explored. However, their views are crucial, given their central role in supporting child development.
- EYPs and public health policymakers have voiced concern about how increasing the use of IEDs might impact children's social and physical development by limiting other forms of play. Nonetheless, IEDs can be valuable resources for EYPs to use in the classroom for educational purposes.
- More guidance is needed to support EYPs, including developing interventions using a whole‐system approach.

## 1 Introduction

It has been reported that in the United Kingdom, 99% of all children went online using laptops, tablets and mobile phones in 2021 and 17% of children aged 3–4 have a mobile phone (Ofcom 2023). Some research also demonstrates that 67.2% of children aged 2–4 years use interactive media (Guedes et al. 2019). Nevertheless, evidence of the impact of interactive electronic devices (IEDs), such as tablets and smartphones, is limited and contradictory (Herodotou 2018; Chindamo et al. 2019).

Earlier research has found a significant association between the duration of a child's smartphone and tablet use and positive gross (Chaibal and Chaiyakul 2022) and fine motor development (Moon et al. 2018). However, contrasting results on language development showed negative results (Moon et al. 2018). Likewise, there is evidence that excessive smartphone use is associated with poorer mental health in children (Sohn et al. 2019) and increased risk of ocular symptoms such as myopia (Al‐Mohtaseb et al. 2021).

A systematic review of qualitative studies has explored parents' perceptions of screen viewing, concluding that there were ‘concerns and confusion’ towards managing screen time and highlighting the need for interventions (Chong, Teo, and Shorey 2023). However, only a few studies have focused on early years practitioners (EYPs), and most tend to focus on how IEDs can be implemented in class (Aljaberi 2021; Nikolopoulou and Gialamas Gialamas 2015; Plowman, Stephen, and McPake 2010). Early years educators see the use of digital tools as valuable, helping to enhance curricular integration, expand children's awareness of the world, help develop operational skills and foster a propensity to learn (Nikolopoulou and Gialamas 2015; Plowman, Stephen, and McPake 2010). Nevertheless, they caution against the use for ‘free play’ (Nikolopoulou and Gialamas 2015). Equally, no studies have investigated the views of EYPs or policymakers on children's everyday use of IEDs, noting that establishing relationships with policymakers has been identified as a critical factor in encouraging their use of evidence (Oliver et al. 2014). Therefore, to contribute to knowledge in this field, this study aimed to explore the perspectives of EYPs and public health consultants (PHC) on the benefits and harms of the use of IEDs by young children (aged 3–5 years).

### 1.1 Methods

This study followed the Standards for Reporting Qualitative Research (SRQR) (O'Brien et al. 2014). Ethical approval was obtained from the University of Huddersfield (SREIC/2021/103). Participants were contacted via email, provided with the study information and assured of their rights as participants.

### 1.2 Participants and Sampling Strategy

Participants were recruited using purposive sampling. We had support from gatekeepers from the local government who directed us to nurseries in the area in Yorkshire, England. EYPs were recruited from two nurseries, one located in a low‐income area and the other in a high‐income area. We used the English Index of Multiple Deprivation (IMD) to help identify the most and least deprived areas in England, with the lowest scores showing the most disadvantaged and the highest scores the least disadvantaged (McLennan et al. 2019). The first nursery's IMD score was 61.28 in the 5th quintile (least deprived), and the second nursery's IMD score was 9.43 in the 2nd quintile (most deprived). Early years PHCs were recruited from a local authority in the Yorkshire region via email. All participants were female, and their occupational roles and years of experience are described in Table 1.

**TABLE 1:** Participant characteristics.

Occupational role	Years of experience
Early years practitioners	
1 Deputy head teacher	19+ years
2 Early years practitioner	22+ years
3 Assistant head teacher	Not disclosed
4 Nursery senior leaders practitioner	25+ years
Public health consultants	
5 Government early years public health consultant	10+ years
6 Government early years public health consultant	Not disclosed

### 1.3 Early Years Settings and Study Design

We visited early years nurseries to conduct face‐to‐face qualitative interviews with EYPs (*n* = 4). Following the EYPs interviews, we conducted online interviews via Microsoft Teams with two PHCs. We used the findings from the EYPs interviews to inform the interview topic guide with PHCs. All interviews were conducted by two members of the research team (L.A. and M.D.).

In the United Kingdom, nurseries provide care for children aged 6 weeks to 5 years and primarily focus on childcare and follow the early years foundation stage curriculum, which provides an age‐appropriate framework for learning (Gov.uk 2023). Early years PHCs work in the local government to support the council's statutory duties in childcare provision, including working with early years providers and EYPs to ensure the delivery of high‐quality childcare and early years education (Local Government Association 2023).

### 1.4 Context of Interviews

EYPs' experience and perspective on how using IEDs affects children's learning, behaviour, physical and psychological development were captured through semi‐structured interviews. The interview topic guide can be found in Data S1. The interviews ranged from 20 to 60 min and were audio‐recorded and stored on a password‐protected hard drive.

### 1.5 Data Analysis

The audio recordings of the interviews were transcribed verbatim to avoid interpretation bias. Face‐to‐face interviews were transcribed using word processing software, whereas the online interview was transcribed using Teams' automatic transcription feature. The first author removed any identifying information before importing the transcripts into NVivo 12, where they were reviewed by the last author prior to analysis.

We used thematic analysis to identify themes and patterns (Braun and Clarke 2006). Our analytical strategy was inductive by nature. To reduce the potential for bias, transcripts were independently coded, our interpretations were discussed and reviewed with the research team, and any discrepancies were resolved via discussion.

We analysed the data from the EYPs and the PHCs as separate entities. First, we analysed the EYP's data and generated primary conclusions. We believe that data saturation was reached after the four interviews since themes that emerged from the analysis of EYPs data provided sufficient detail and offered good variety (Patton 2014). Preliminary themes from the EPY's interviews were then used to inform the interview topic guide with PHCs (Data S1), arousing further themes. Although two interviews with PHCs might not be deemed sufficient to reach saturation, they were adequate to provide enough information power (Sim et al. 2018) and cover the breadth and focus of our research question by offering valuable insights into the PHC's perspectives on using IEDs (Braun and Clarke 2021).

## 2 Results

Themes and subthemes for EYP's data are presented in Table 2, together with the PHC theme, for comparison purposes. Further exemplar quotations can be found in Data S2.

**TABLE 2:** Themes and sub‐themes found from reflective thematic analysis of EYP interview data.

Themes EYP	Sub‐theme EYP	Themes PHC
Reasons for increased use of IEDs	Management of daily tasks, work commitments and busy parent schedules Generational changes and advances in technology Parents' attitudes and habits surrounding IEDs	IEDs as a substitute for play and parents' attitudes
IED's impact on communication, behaviour and learning	IED impact on communication IED impact on child's behaviour and development IED as a practical tool for learning	Impact of IED on child communication and social development Impact of IED on motor development and physical activity IED use in nursery setting
Collaborative approach—EYPs and parents	Accountability in education Supporting relationships	Intervention development
Educating parents and teachers on the use of IEDs by the child	Parents' knowledge concerning the impact of IEDs Interventions for EYPs and parents	

### 2.1 Reasons for Increased Use of IEDs

#### 2.1.1 Management of Daily Tasks, Working Commitments and Busy Parent Schedules

EYPs in both nursery settings reported a perception of increased use due to work commitments, time management skills and first‐hand experiences.

It's just that there's so many factors you know about people's lifestyles. Depending on how much time they've got, how many hours they work, the age of the children, their own experiences, and their own computer use. Erm, yeah, it is tricky. (High‐income area, EYP)

EYPs showed mindful reasoning towards the necessity of needing to use IEDs with children for parents who are single, outlining that this is becoming a routine part of day‐to‐day family life and expressing acceptance towards the increase in use.

If they've been, you know, had a hectic day, but maybe that half an hour is. Oh, I'll unpack the bags and get the packed lunches done as well, but generally, you know, she doesn't have a lot of support and she's actually very mindful of it, so I think it it's really hard to say isn't. (Low‐income area, EYP)

#### 2.1.2 Generational Changes and Advances in Technology

EYPs across both nurseries discussed how technology had significantly changed during their careers.

I think just because of the way that the world is going. (High‐income area, EYP)

Comparisons were made between ‘old box computers’ and modern‐day technology, highlighting how children interacted with wooden toys by swiping their fingers across a wooden block, as though using an iPad or similar interactive device.

We had the old box computers … Now, with what is it called the touch screen and the tablet? But I feel like it's every child automatically expects things to happen by touching them … We've got wooden bricks, and children touch those swipe them, and they think something is going to happen. (Low‐income area, EYP)

#### 2.1.3 Parents' Attitudes and Habits Surrounding IEDs

EYPs across both nurseries expressed discomfort with the habits of some parents when using IEDs themselves, mentioning how it can cause important in‐person information to parents during school times to be disrupted.

I think it's frustrating for us as teachers as well because we need that time just to communicate with the parent and when and I've got a parent on an afternoon, and I said, can you and I … I said please, can you put your phone down? I need to talk to you orrr … it's my life, it's my life. And it's like it doesn't. It's not important, yeah, so I don't know. I think … is it people's perceptions of what life is? Has it become such a norm? (Low‐Income area, EYP)

EYPs empathise with how EYPs value and respect an in‐person interaction with parents and believe this is meaningful.

### 2.2 IED's Impact on Communication, Behaviour and Learning

#### 2.2.1 IED Impact on Communication

There was a belief among most participants that language changes could be due to influences like YouTube, meaning accents and vocabulary from around the world.

Maybe a little bit of language as well, and you see, sometimes suppose this is something that I have noticed then. So, when they're playing imaginatively, they kind of put on an accent … almost Americanized. (High‐income area, EYP)

For children with special educational needs (SEN), IEDs were viewed to have positive outcomes on learning vocabulary.

We have some of our special educational needs children who have more vocabulary than you would expect for children of their level of development, erm, and nearly all of that's been learned through devices. (Low‐income, EPY)

Learning via games and apps may offer a way of embedding vocabulary with children, which leads to the potential for developing contextual learning in the future.

I think he, I don't know, the brain maybe memorises things when it's repeated in, you know, in a stimulating way, like with a device, maybe more so than an actual social interaction with some children. (Low‐income area, EYP)

Conversely, communication might also be negatively impacting between EYPs and parents (i.e., ‘Parents attitudes and habits surrounding IEDs’ theme) and between parents and their children.

#### 2.2.2 IED Impact on Children's Behaviour and Development

Insights were offered on the importance of being aware of how IEDs might have a negative influence on child development and behaviour.

I'm finding children are coming in with poorer communication skills and physical skills where you know what I used to think 20 years back children had these things automatically. (Low‐income area, EYP)

EYP suggested that IED use can negatively affect a child's temperament as the quick interface of an iPad leads children to have higher expectations and less patience.

But this way, because they're just touching the screen, they expect something to happen instantly. And then they get frustrated …. Cause so if something does not happen instantly then just randomly touch things. (Low‐income area, EYP)

EYPs from both nurseries noted a reduced ability of child self‐regulation and coping behaviours that appear to have become more pronounced in recent years, and they suggested links to the increased use of IEDs.

#### 2.2.3 IED as a Practical Tool for Learning

Some EYPs believe that IEDs need to be considered only as an adjunct learning resource used in combination with other resources and be led by adults with a sense of reasonable control and careful attention towards what content children can access and monitor their use.

Yeah, I mean the younger ones, might you know if they're learning a new song or something, they might put a song on and, it's probably more adult lead activities rather than, you know, giving them an iPad and letting them play on a game. (High‐income area, EYP)

EPYs offered insights highlighting how IEDs could assist learning, including illustrating learning through videos or educational games. However, it should not be a substitute for social learning without IEDs.

We've got a few phonics games we play on there, but we play as a group. You know, erm, socially, if children have asked us a question or I've got, you know, curious about something, we might use Google and search and learn more, so we model that so we're not against exposure to technology. I guess we just don't want it to be a distraction from active social learning, which you know is obviously one of our big focuses is at nursery. (Low income, EYP)

### 2.3 Collaborative Approach: EYPs and Parents

#### 2.3.1 Accountability in Education

EYPs expressed how accountability with parents is essential to promoting education and how the environment could be very important.

It's such a rich learning environment we have here. If they have that experience at home, yeah, then it will limit them if that is always going to be their first choice. (Low income, EYP)

One EYP explained that some children entering nursery school are less school‐ready than they were, inferring that interaction between the parent and child has lately been impacted by IEDs.

Some of them are not as school ready as you would want them to be, but you only have that certain amount of time to work with them, and I find when you work with parents that do want to work … It works because the child is getting the impact from both sides …. (Low‐income area, EYP)

… For example, toilet training, I've had children where I can help them, but the parents don't, so as soon as they take them home, they're putting nappies on them. So that's having an impact on my work. (Low‐income area, EYP)

#### 2.3.2 Supporting Relationships

Some EYPs expressed how using IEDs allowed them to maintain a connection with the parents and children during times when they were absent from nursery, especially during COVID‐19.

It wasn't as such formal learning. But we just felt like we wanted to support our families. (High‐income area, EYP)

As a result, IEDs were beneficial for facilitating support, which improved relationships between parents, EYPs and children, illustrating how technology can be both advantageous and but also disadvantageous (‘Parents attitudes and habits surrounding IEDs’ theme).

### 2.4 Educating Parents and EYPs on the Use of IEDs by the Child

#### 2.4.1 Parents' Knowledge Concerning the Impact of IEDs

The EYPs were mindful that parents did have an intuitive base knowledge of the impacts of IED overuse. However, busy schedules and daily life often led to distractions and mild forgetfulness towards the topic.

… And then maybe those parents. I don't know, kind of forget or it's not at the forefront of their mind. You know the consequences that they were maybe worried about to begin with. (High‐income area, EYP)

I think that those boundaries that may be initially set erm broadened and widened as time goes on. I think that's probably what happens …. (High‐income area, EYP)

They implied how a refocus on the topic would be beneficial for both parents and EYPs to improve awareness, with additional discussions around how to position messages about the impacts of IEDs.

#### 2.4.2 Interventions for EYPs and Parents

All EYPs saw potential in producing new evidence and offered ideas on approaching new interventions.

Cause as a parent myself, if somebody told me that, I think wow, 'cause it really hits you like you know this is the impact the negative impact this technology is having on my child, and until somebody brings that to the forefront, you don't always, yeah, realise. (Low‐income, EYP)

All opted for collaboration between parents and EYPs on intervention development and how EYPs should support parents.

Think for early years practitioners we also need to help them (parents) to learn how to use this and how long and content and so on. (High‐income, EYP)

#### 2.4.3 Reflecting on the EYPs Views With PHCs

The following section reflects the opinions of PHCs and their views on the findings from the EYP interviews. More exemplar quotes are found in Data S2.

#### 2.4.4 IEDs as a Substitute for Play and parents' Attitudes

When PCHs were asked how parents manage children's use of IEDs, they reported that parents should be encouraged to take children outdoors.

But you know, a lot of people have back gardens, and they could go out into parks, and they could go out into streets and stuff like that. It is not about spending loads of money on children it's about having that time, and I just don't think parents have that time anymore. They just use the iPad, you know, because it's easier. (PHC, 1)

PHCs agreed with EYPs that parents have been using IEDs excessively, and this is affecting the child's development. However, they also attribute the lack of child's play to ‘adult barriers’.

Yeah, and I could also link to the restrictions of play because obviously, there seem to be fewer play opportunities for children, and a lot of that sort of probably down to like adult barriers …. (PHC, 1)

They agreed with EYPs on how the content watched and parental control of the children's devices showed an awareness of the difficulty associated with teaching children about the risks related to IEDs. They also reported the different approaches used at home or in early years settings.

So again, it's about the context of what is happening. You know, wherever they should. Because those children get free access to iPads and mobile phones at home, so it needs restricting within that nursery environment. (PHC, 1)

#### 2.4.5 Impact of IED on Child Communication and Social Development

PHCs raised concerns about the overuse of IEDs by young children and the impact this might have on play‐based social development opportunities and possible long‐term effects.

When they're using their iPads when they're that age because they're not developing those social communication skills. They're going to struggle, you know, doing things for themselves, finding out learning mistakes …. That will provide a longer‐term problem. (PHC, 1)

PHCs agree with EYPs that the lack of social interactions resulting from the overuse of IEDs has a potential negative impact on the child's communication and social skills.

… if they're spending a lot of time on the screen, then they're not necessarily socially interacting with their peers, so things like taking turns and even just going back and forth in conversations. The serve and return in terms of speech and language listening skills again can be negatively impacted. (PHC, 2)

They also acknowledged how some children appeared not to be absorbing information from their surroundings when engaged in the IEDs, affecting their sensory development.

… so they actually tune out they're not listening to what is being said around, so they're not picking up on that conversation, but they're also maybe not using conversation either and affecting their ability to concentrate because they'll concentrate very much on the screen but not concentrate on other things. (PHC, 2)

#### 2.4.6 Impact of IED on Motor Development and Physical Activity

There was a particular concern from PHCs on motor development, which was not raised by EYPs.

So, they were spending that much time on the iPads or phones or whatever that they were missing out on those basic essential skills like balance, coordination, and spatial awareness … It was shocking, really, because like, at that age now, they should be able to be able to go out and run around and not walk or bump into each other like they are doing. (PHC, 1)

They are especially worried about the effects that IED use has on children playing outdoors, taking risks and being active.

Again, that is because they're not going out and playing in that garden or whatever or being allowed to take those risks like balancing and doing stuff that children should be doing to learn all these skills and then again, where does that lead them in future? (PHC, 1)

#### 2.4.7 IED Use in Nursery Settings

Some PHCs are concerned that EYPs rely too much on iPads to deliver the session, which could affect their practical experience in some aspects of learning.

I found that teachers relied a lot on iPads, to deliver lessons. So in terms of like going to YouTube … the teachers seem to find it easier to do it online because it is less hassle than having to set up resources tools to get the children all sorted out to do that practical lesson. (PHC, 2)

They further reported that practical lessons would teach children other skills, such as social and motor skills, that could not be learned using iPads.

But actually, when you gave them for Jigsaw, they were unable to put those pieces together. Erm because they couldn't physically do it, you know they have those fine motor skills. (PHC, 2)

The PHCs believe that relying on IEDs for learning is due to time pressure to cover the curriculum.

Because they wanted those students to meet those objectives … So, and because of time constraints, I think they found it just easier to go on the iPads, get it done, dusted and the lessons finished. (PHC, 1)

On the other hand, PHCs also believe that some EYPs might have preconceived ideas about IED use because of their own lack of knowledge, therefore seeing it as unfavourable and not implemented in class.

And I think some of the drawbacks are, is that some of the adults working with young children are maybe not as familiar with the technology as the children, and perhaps see it as a negative. (PHC, 2)

#### 2.4.8 Intervention Development

The PHCs believed that using IEDs should be learned as other essential skills that you teach to children in their early life.

It's like teaching children to cross the road. We teach them to cross the road safely. We teach them to use roads, pavements, et cetera. In a safe way, that's part of learning, and I think interactive technology needs to be seen in the same way. (PHC, 1)

We have then reported the collaborative approach to deliver the intervention as suggested by EYPs. They added the importance of verifying learning and performing an evaluation and the importance of consistent positive messages delivered regularly across the whole system:

What I'd like you to do is get a bit of feedback on whether that worked. What is the impact? Did you do it? Has it worked out? What did you get from it? Was it a waste of time? (PHC, 1)

So it has to be consistent messages across all. So what are children learning that our parents are being given? What do the settings know, and how do we build on those different stages? (PHC, 2)

Due to the ever‐changing nature of technology, PHCs recognise the need for new interventions implemented in early years settings to be adaptable and flexible in their approach.

As technology develops as things change, they need to feel that they are confident to keep up to date and abreast of that. (PHC, 2)

#### 2.4.9 Comparison Themes for EYPs and PHCs

EYPs and PHCs both agree that parents' attitudes have an impact on the use of IEDs. EYPs are concerned about parents' own use of IEDs, whereas PHCs are more worried about the implications of IEDs being used as a substitute for traditional play. They agree that IEDs might harm child communication. However, PHCs also focus on the negative impact on social and physical development. EYPs and PHCs see IEDs as helpful learning tools, particularly in areas that cannot be easily illustrated in a classroom setting. However, PHCs are concerned about the overuse of IEDs and how this could affect practical experiences that support the development of other skills. Finally, they both agree that interventions are needed using a holistic approach involving the child, parents and nursery, with PHCs emphasising the importance of conducting evaluations as part of these interventions.

## 3 Discussion

This study sought to report on the views of EYPs and PHCs concerning the use of IEDs by young children, including behaviour, development and intervention needs. EYPs attribute the conditions surrounding the rising use of IEDs by young children to the approach parents use to juggle their everyday obligations and work commitments. They recognised that these are generational changes brought by the advances in technology but also acknowledged that parents' use of IEDs affects interaction with the EYPs and children. This study found that the most prominent concerns related to IED overuse are communication skills, self‐regulation and physical activity levels, as underlined by EYPs and PHCs. However, IEDs may be advantageously and successfully employed as a teaching tool to improve parent–teacher communication in an educational setting, with EYPs recalling how IEDs were beneficial during the COVID‐19 lockdown. EYPs and PHCs emphasised the need for increased knowledge and awareness of IED use among children, parents and EYPs. They also encouraged innovative and interactive intervention approaches to inform IED use.

The literature shows that children's screen‐viewing behaviour has rapidly evolved. Studies dated from 2016 to 2017 (Bentley, Turner, and Jago 2016; Kostyrka‐Allchorne, Cooper, and Simpson 2017) indicated that TV viewing was the favourite media type for young children. However, more recent studies (Guedes et al. 2019; Geurts et al. 2021) and the UK's communications regulator, Ofcom (2022), show that ‘portable devices’, such as IEDs, are now the most common type of media used by children of all ages. The interactive and portable characteristics of the devices appear to have influenced a change in behaviour, and it is essential to look at how this influences the development of young children.

Several studies have explored parents' opinions regarding using IEDs (Bentley, Turner, and Jago 2016; Kostyrka‐Allchorne, Cooper, and Simpson 2017; Hood et al. 2021; Geurts et al. 2021); however, no studies that have looked at the perspective of EYPs regarding how IEDs impact young children's lives. Some studies focused on applying IEDs as a learning tool in class (Aljaberi 2021; Plowman, Stephen, and McPake 2010;Nikolopoulou 2021). Preschool teachers perceived that the device might support learning through interactive opportunities. However, it might also restrict ‘hands‐on’ experience and child concentration (Nikolopoulou 2021), which coincides with the views of PHCs in our study. Together with parents, EYPs play a vital role in supporting the development of young children (Allen et al. 2019). Therefore, their viewpoints are essential to comprehend how IEDs as a phenomenon are influencing the lives of young children.

It has been reported that parents' screen‐viewing habits can influence children's behaviour (Thompson et al. 2017). EYPs in this study expressed concern that parents' usage of IEDs may harm the interaction between parents and children. An ethnographic study that observed parent–child interaction in the playground noted that parental use of mobile phones was linked to parental disengagement, which had safety and emotional consequences giving children less opportunity for social learning (Lemish, Elias, and Floegel 2020). Similarly, a scoping review (Kivijärvi et al. 2005) noticed an association between parental mobile device use and parental sensitivity and responsiveness, negatively impacting parent–child attachment and affecting the child's self‐regulatory capacity.

EYPs and PHCs in our study confirmed the findings of a previous study (Bentley, Turner, and Jago 2016) by reporting that parents may be utilising IEDs as ways to ‘babysit’ or manage children. However, using IEDs frequently may prevent children from other forms of playing (i.e., toys and outdoors), which has benefits compared to IEDs, particularly in terms of social skills (Li, Hestenes, and Wang 2016), which agrees with the views on PHCs in our study. EYPs also noted that IED use may have impacted children's self‐regulation and underlined the recent decline in school readiness in the United Kingdom (YouGov 2023).

On the other hand, EYPs and PHCs also reported that IEDs could be a ‘lifesaver’ and a vital educational tool, particularly during the COVID‐19 pandemic. Other studies also report that parents believe that IEDs are a superior educational tool to TV viewing (Jago et al. 2014; Kostyrka‐Allchorne, Cooper, and Simpson 2017). It has been argued that IEDs can enhance language learning in young children when content is co‐viewed and discussed with parents (Walter‐Laager et al. 2017), which was an aspect also positively raised by the EYPs when highlighting an adult‐led approach to IED use. A systematic review supported the positive effect of tablets on young children's (2–5 years) learning and development, particularly in literacy, mathematics, science, problem‐solving and self‐efficacy. However, the review was limited to observational studies (Herodotou 2018).

Our research found that EYPs and PHCs believed interventions around the use of IEDs to be valuable and necessary. They proposed a holistic approach, with messages involving children, parents, EYPs and PHCs. There were also discussions on the need for interaction and constant adaptation due to continuous technological change.

Guidelines on how IEDs should be used during early childhood are still vague due to limited evidence in the area. The World Health Organisation advises that children under the age of 5 should spend 1 h or less, whereas children under the age of 2 should spend no time at all on‐screen devices (World Health Organisation 2019). Others, like the UK government, provide recommendations for early years settings, focusing on online safeguarding for children and professionals (UK Council for Internet Safety 2019). Similarly, although some guidelines provide recommendations on the duration of screen use by children (American Academy of Child& Adolescent Psychiatrist 2024; Canadian Paediatric Society 2017), these are not based on solid evidence, with the UK recommendations preferring not to set threshold and instead looking at individual needs and balancing physical and social activities and sleep (Viner, Davie, and Firth 2019).

## 4 Strengths and Weaknesses

Our study was constrained by the sample, which constituted two EYPs in each early year's setting (four in total) and two PHCs and included only female perspectives. However, our participants had a wealth of experience in the education sector (around 20 years) and came from diverse backgrounds. The interviews with EYPs indicated that we reached saturation as no new themes have emerged from the data. However, the two interviews with PHCs might not have reached saturation, but it was enough to identify prevalent themes and provide ‘information power’. The same researchers who collected the data also analysed it, which might have increased the risk of bias.

## 5 Conclusion

The study provides new evidence that emphasises the experiences of early childhood educators, highlighting potential reasons for the rise in IED use and identifying the double‐edged nature of IED use and both their positive and negative impacts on young children's development. Although the study does not offer a guideline on how IED should be used at home and in early years settings, it provides a clear message that this is an area of uncertainty but also of great need. Future research should investigate the causal relationship between using IEDs and outcomes related to young children's development and inform guidance to maximise the benefits and minimise the risks (Straker et al. 2018). EYPs and PHC ask for a holistic approach which could involve children, parents, early years settings and the government to guide young children on IED use.

## Author Contributions

**Liane B. Azevedo:** conceptualisation, investigation, writing – original draft, methodology, writing – review and editing, formal analysis, project administration, supervision. **Megan Downes:** writing – original draft, methodology, writing – review and editing, formal analysis. **Sara Eastburn:** formal analysis, writing – review and editing, writing – original draft. **Jane Covell:** conceptualisation, writing – review and editing, methodology. **Paul Bissell:** conceptualisation, formal analysis, writing – original draft, writing – review and editing.

## Ethics Statement

Ethical approval was obtained from the University of Huddersfield (SREIC/2021/103).

## Consent

Participants were provided with the study information, and all participants signed an informed consent form.

## Conflicts of Interest

The authors declare no conflicts of interest.

## Supporting information


**Data S1.** Topic guides

**Data S2.** Further exemplar quotations

## Data Availability

The data that support the findings of this study are provided in Data S2.
