# Supplementary material for: Early Years Practitioners' and Public Health Consultants' Perspectives on the Use of Interactive Electronic Devices in Young Children: A Qualitative Study
Source: Child Care Health Dev. 2025 Jan 12;51(1):e70022. doi: 10.1111/cch.70022 (PMC11725388; doi:10.1111/cch.70022)
Supplement: Supplementary file 1 — Data S1. Topic guides [file CCH-51-e70022-s002.docx]

**Supplementary file 1: Topic guides**

| **Topic Guide –Early Years Practitioners** |
| --- |
| 1. What are your views of young children’s use of interactive technology?  2. Can you talk to me about some of your views on the benefits and harms?  3. What do you think is the role of interactive technology in young children education?  4. What do you think is the role of interactive technology in families’ life?  5. Do you think interactive technology can benefit and/or harm young children’s development (physical, mental or emotional)?  6. Do you feel there is a need to develop interventions to guide nurseries on the use of technology? How do you think this intervention could look like?  7. Do you there is a need to develop interventions to guide parents on the use of technology? How do you think this intervention could look like?  8. Do we need evidence-based guidelines to guide teachers and parents on the use of interactive technology?  9. Technology is fast-pacing and quick changing. Do you have any ideas on how we could adapt to the constant changes. |

| **Topic Guide –Public health consultants** |
| --- |
| 1. What do you think is the role of IED in young children education? 2. How do you think IED can impact on young children’s development (physical, cognitive and social)? 3. What do you think is impact of IED in families’ life? 4. Do we need evidence-based guidelines to guide early years practitioners and parents on the use of IED? 5. Do you think there is a need to develop interventions to guide childcares and/or parents on the use of technology? 6. What could be the key ingredients of this intervention (based on other public health interventions for this age group)?   **Share findings (once we finish the interview)**  **Impact on creativity, learning, motor skills**  **Question**  Do you agree? How could we use IED to improve creativity and learning? What about motor skills?  **Use of free video sharing platforms**  **Question**  What are your perceptions about video sharing platforms for children?  **Question**  Do you think IEDs have been used as baby sitting? What is the short- and long-term impact of this use?  **IEDs vs play**  **Question**  Do you feel that IEDs have been replaced by usual toys? What do you think are the positive and negative impact of this?  **Parent- child interaction**  **Question**  What are your views on parent-use of IEDs and the impact on child and family cohension?  **Dose and when to introduce**  **Question**  What are your views about duration and when in the child life cycle this should be introduced? |
