# Supplementary material for: Early Years Practitioners' and Public Health Consultants' Perspectives on the Use of Interactive Electronic Devices in Young Children: A Qualitative Study
Source: Child Care Health Dev. 2025 Jan 12;51(1):e70022. doi: 10.1111/cch.70022 (PMC11725388; doi:10.1111/cch.70022)
Supplement: Supplementary file 2 — Data S2. Further exemplar quotations [file CCH-51-e70022-s001.docx]

**Supplementary file 2: Further exemplar quotations**

**List of Abbreviations**

| **Phrase** | **Abbreviation** |
| --- | --- |
| Interactive electronic devices | IED |
| Public Health Consultant | PHC |
| Early Years Practitioners | EYP |
| Special Educational Needs | SEN |

1. **Further quotations early years practitioners**
2. **Reasons for increased use**

**Management of daily tasks, working commitments and busy parent schedules.**

“It's just that there are so many factors you know about people's lifestyles. It depends on how much time they've got, how many hours they work, the age of the children, their own experiences, and their own computer use. Erm, yeah, it is tricky” (High-income, EYP).

“But then single parents, it's my... Erm my friends' children who have the iPads for half an hour after school as a bit of calming activity. That is, she's a single mum, so maybe that half an hour, the children do enjoy it, and it does calm them down. If they've been, you know, had a hectic day, but maybe that half an hour is. Oh, I'll unpack the bags and get the packed lunches done as well, but generally, you know, she doesn't have a lot of support, and she's actually very mindful of it, so I think it it's really hard to say isn't” (Low-income, EYP).

“I suppose parents may have been busy working from home, so it may have been that they may have had to have that option with the iPad and things like that” (High-income, EYP).

**Generational Changes and advances in technology.**

“I think just because of the way that the world is going” (High-income, EYP).

“We have some of our special educational needs children who have more vocabulary than you would expect for children of their level of development erm and nearly all of that's been learned through devices” (Low-income, EPY).

“When I see over a length of time when I first initially started, we hardly had anything. We had the old box computers. With the keyboard, it was very much like pressing a key and seeing something happening on the screen, and those children there were certain children that weren't interested in it and certain children that went, and it was very much of using the key using the mouse and hand-eye coordination. So, when I moved the mouse here, something was going to happen on the screen, and then I needed to press the button on the mouse, so there was hand-eye coordination going on at that time. Now what is it called when they're touching what’s it’s called the touch screen and the tablet? But I feel like it's every child automatically expects things to happen by touching them. Cause sometimes we've got. We've got block players. We've got wooden bricks and children touch, those swipe them, and they think something is going to happen so my personal feelings on it is it's not good to have all the time when we did have the old computers they were learning hand and eye coordination in the fact that they were using a mouse but this way because they're just touching the screen, they expect something to happen instantly” (Low-income, EYP).

**Parents’ attitudes and habits surrounding IED.**

“Yeah, that we cannot cut one conversation off before and then in his language he said to his wife that I need to talk to the teacher, so he put it down and then spoke to me... a quick conversation. And then, as he walked, he picked it back up and put it on his ear. To think that children must feel frustrated when they see their parents and the parents don't give attention to them. Like, of course, they have spent the whole day at the nursery. They might want to hold hands?” (Low-Income, EYP).

“I think I'm kind of opposed to a lot of it just because, in my view, children do get too much of it, and it becomes a bit of a babysitter quite often. Erm, I have some family members who were very dependent upon, like on the iPads and the phones and things like that and erm, it causes a lot of tantrums and erm, I just think too much dependence. You know, whilst they eat, to sit dinner, to sit down and eat dinner, they must have the iPad there” (High-income, EYP).

“I think it's frustrating for us as teachers as well because we need that time just to communicate with the parent and when and I've got a parent on an afternoon, and I said, can you and I …I said please, can you put your phone down? I need to talk to you orrr… it's my life, it's my life. And it's like it doesn't. It's not important, yeah, so I don't know. I think…is it people’s perceptions of what life is? Has it become such a norm?” (Low-Income, EYP).

1. **Impact on communication, behaviour, and learning**

**IED impact on communication**

“Maybe a little bit of language as well, and you see, sometimes suppose this is something that I have noticed then. So, when they're playing imaginatively, they kind of put on an accent... almost Americanized.” (High-income, EYP)

“A lot are children who watch not, just with interactive devices but with TV, are using a lot of like very American language and vocab.” (Low-income, EYP )

“He’ll ask for, like, rather than a biscuit, ask for a cookie, and you know things like that where you're just like, oh, this is interesting. Things like that. I can't think of any other examples right now. But there's quite a few of them that pop up... like garbage” (Low-income, EYP).

“Well, it is a positive because we have the words that we have, and hopefully then they will learn context and how to use them, but yeah, it definitely. I think he, I don't know, the brain may memorise things when they're repeated in a stimulating way, like with a device, maybe more so than an actual social interaction with some children” (Low-income, EYP).

**IED impact on child’s behaviour and development**

“I think it's just all about moderation, and it's not forgetting that you know it can interrupt their development” (High-income, EYP).

“I think technology could be having an impact on it, but I'm finding children are coming in with poorer communication skills and physical skills where you know what I used to think 20 years back children had these things automatically” (Low-income, EYP).

“My personal feelings about it are that it's not good to have all the time when we did have the old computers. They were learning hand and eye coordination in the fact that they were using a mouse. But this way, because they're just touching the screen, they expect something to happen instantly. And then they get frustrated. They do. They do get frustrated. Cause so if something does not happen instantly then just randomly touch things” (Low-income, EYP).

**IED as a practical tool for learning**

“I think they set the time, like just probably not the time. I don't know if there's a way of doing that on the app, but just themselves, you know, like monitoring how long they're spending on there. Erm, and yes, and yeah, certainly some of the parents kind of check which apps they think are appropriate. Or, you know, they only have apps on there that they deem to be educational” (Low-income, EYP).

“I'd say so. We don't really have…Yeah, I mean the younger ones, might you know if they're learning a new song or something, they might put a song on and, it's probably more adult lead activities rather than, you know, giving them an iPad and letting them play on a game” (High-income, EYP).

“We've got a few phonics games we play on there, but we play as a group. You know, erm, socially, if children have asked us a question or I've got, you know, curious about something, we might use Google and search and learn more, so we model that so we're not against exposure to technology. I guess we just don't want it to be a distraction from active social learning, which you know is obviously one of our big focuses is at nursery” (Low income, EYP).

“Erm, I think some teachers would benefit from it and erm but then it's difficult to say here because I think. Because the head teacher and I are kind of against the overuse of technology in the rooms, everyone else is just kind of Uh, understands that and accepts it. But even actually, there are times when our Interactive Whiteboard is in the community room. For example, if we're actively planning a session, we'll go and use that. But sometimes, at the end of the day, some teachers will put nursery rhymes on the screen for the children, which obviously is a fantastic thing, but I would rather them sing the nursery rhymes rather than just be on the screen. But sometimes, logistically, that’s not an option if the adults are at the door, seeing parents. So, erm yeah, I wonder if it wasn't controlled here, if some would put screens on a little bit more. So yeah, maybe developing the educator's understandings as well as the pros and cons, and consequences” (Low-income, EPY).

1. **Collaboration between teachers and parents**

**Accountability in education**

“It's such a rich learning environment we have here if they have that experience at home, yeah, then will limit them if that is always going to be their first choice. So we still expose them to like, we model using the iPads to take photos, you know, record sounds” (Low income, EYP).

“Some of them are not as school ready as you would want them to be, but you only have that certain amount of time to work with them, and I find when you work with parents that do want to work…It works because the child is getting the impact from both sides. For example, toilet training, I've had children where I can help them, but the parents don't, so as soon as they take them home, they're putting nappies on them. So that's having an impact on my work, whereas I'm having to take time out of my teaching to take them to the toilet” (Low-income, EYP).

**Supporting relationships.**

“It's because parents are so busy on their phones because we see it and we've got...We've got so many signs up in the nursery on both sides of the entrances. Please do not use your mobile phone when you are picking your child up. There are certain parents that will listen and certain ones they don’t... they are not bothered” (Low-income, EPY).

“And so, we do often take photos and things, and the children, particularly when they get a little bit older. If they have done something or they dressed up in something, they might want to show Mummy or Daddy. Or we can send that down back to them so they can see. And so, we do use it like that, but in terms of the children's access to the nursery setting, it's quite minimal, really.” (High-income, EYP).

“It wasn't as such formal learning. It was modesty because we actually closed for three months because we had no key children, and that needed, you know, our service job in the three months or from March 2020 to June 2020 at the end of March, and we did close. But we just felt like we wanted to support our families because we didn't know if they'd got, you know, grandparents and friends. You know, they could have caught them, but, uh, moved up here with our families moving from London. They don't know people, so it was more of a way of just giving them that support” (High-income, EYP).

“You know it, it was just a long period of time for them, and so the stories and the songs that we sang to them on the videos, and we showed them to the parents. It's all optional. It wasn't like a set, even a bit like schools. Maybe it was just too really... just to keep the bonds” (High-income, EYP).

1. **Educating parents and teachers for the benefit of the child**

**Parent knowledge concerning the impact of IEDs**

“Erm yeah, kind of that rope learning. I think it's probably can be used to be in a beneficial way. But it can't be used in isolation. I think that then needs to be expanded and used with, you know, with other humans and in context for it to have an actual impact. I'd say” (High-income, EYP).

“I’ve got some friends of mine who use iPads, and in children who have been very active all day, you've had lots of social and lots of communication, interaction erm choose to have it for half an hour when they get home because they see it as relaxation, its downtime and those parents' kind of monitor what apps their children are using, so they're kind of... Educational and you know the time that that that that's being used on these restrictive, so I'm not against him completely because I do see people using them that way, but I just think... with certain family members, it can be quite detrimental.” (High-income, EYP).

“I think that those boundaries that may be initially set erm broadened and widened as time goes on. I think that's probably what happens…” (High-income, EYP).

“…And then maybe those parents. I don't know, kind of forget or it's not at the forefront of their mind. You know the consequences that they were maybe worried about to begin with” (High-income, EYP).

**Interventions for EYPs and parents**

“I think the core messages are probably the same, aren't they? For teachers it might be of how to support the parents with better use a bit more. Erm, I think those key messages are the same” (High-income, EYP).

“Think for early years practitioners we also need to help them (parents) to learn how to use this and how long and content and so on.” (High-income, EYP)

“I think. Uhm, like you say, guidance for parents, but they need also, I think, for them to take notice it needs to. They need to understand what the negative consequences could be. You know, with overuse or incorrect use, I think for them to listen, or you know, decide to change how they are using them currently” (High-income, EYP).

“So maybe that could be an intervention that you know teaching parents how, how much, how much and how and what devices yes” (Low-income, EYP).

“Cause as a parent myself, if somebody told me that I think wow, 'cause it really hits you like you know this is the impact the negative impact this technology is having on my child and until somebody brings that to the forefront, you don't always, yeah, realize” (Low-income, EYP)

1. **Further quotations public health consultants**

**IEDs as a substitute for play and parents’ attitudes**

“Physical development and PSED (personal, social and emotional development), and I know we're coming out of COVID and stuff like that, but I'm not being funny. But you know, a lot of people have back gardens, and they could go out into parks, and they could go out into streets and stuff like that. It is not about spending loads of money on children it's about having that time, and I just don't think parents have that time anymore. They just use the iPad, you know, because it's easier” (PHC,1).

“Yeah, and I could also link to the restrictions of play because obviously, you as children have, like, well, you know, there seem to be fewer play opportunities for children, and a lot of that sort of probably down to like adult barriers. So again, because they're not playing out like we used to do if you like, and they're spending more time on the iPads, they're not getting those essential skills like you know, just things like communication and language skills, because they’re not going out on playing, you know. Play dates and stuff like that, there are very few. So, I don't know if it's. If it's the iPad or a mix of both of that, using the iPad or phone or whatever. And the lack of those play opportunities, like you know, because of adult barriers, or you know, potential risks or whatever that adult sees. Erm, and then if it's related to play, it comes down to potential risk or perceived risk by those adults and teachers” (PHC, 1).

“Grand theft auto! That’s it! *laughter* So we have children accessing that because their older brothers were playing it, so they were allowed to sit and watch that. You know, so they're coming back and talking about stuff and you just like… what? You know…” (PHC, 1).

“So again, it's about the context of what is happening. You know, wherever they should. Because those children get free access to iPads and mobile phones at home, so it needs restricting within that nursery environment” (PHC,1)

“Yeah, I think you know, it's like you said, television was the place where the children saw the adverts are sweet cereals and things like that. But now, like I say, depending on what they're on and what the securities on the particular piece of media that they're using, they're exposed to all kinds of marketing, and also not only marketing but the safety and security is not in there. They could be getting all the messages, and we do know that actually some other people who are out there are very clever at sending subliminal messages” (PHC, 2).

**Impact of IED on child communication and social development**

“I think in terms of speech and language, it impacted because they are, uh, they can very easily tune out what's around them, and we know that a lot of languages is caught and not taught, so they actually tune out they're not listening to what is being said around, so they're not picking up on that conversation, but they're also maybe not using conversation either. This affects their ability to concentrate because they'll concentrate very much on the screen but not concentrate on other things, so it can affect their concentration. I think in terms of socialization, and I think it probably does start in early years, but certainly with older children” (PHC, 2).

“When they're using their iPads, when they're that age because they're not developing those social communication skills. They’re going to struggle, you know, doing things for themselves, finding out learning mistakes. All that sort of stuff when that's not happening, I think. That will provide a longer-term problem.”(PHC, 1)

“I'm an outdoor person, so I like children to keep up with nature and all this sort of stuff. And I found that those skills were being missed out, like sharing skills and being able to be, you know, just like linking those social skills that you stop and all that communication language. Because when you are doing hands-on activities, the children speak to each other. You know you found those little team leaders that came out. You found those like saying negotiation skills. You know, those sharing skills”. (PHC1)

“They can certainly be some of those negatives. If you've got children who are spending a long amount of time on interactive media, they may not be getting the physical activity that they need, so they may not be out doing the climbing. They might have, you know, very small. the kind of pins and movements and things like that, but in order to do that, they need the big movements, and we know as adults that if you are using a mouse all the time, you can end up with stress and tension, and so there's the impact on young bodies. Then maybe not getting out and being physically developed and not getting the fresh air which then. Now impact on the sleep, and there's the fact that if they're playing on games that are repetitive activity, but also the fact that there is screen time very close to bedtime can be an issue. In terms of social, uh, and emotional development, if they're spending a lot of time on the screen, then they're not necessarily socially interacting with their peers, so things like. I learned to take turns and even just go back and forth in conversations. The serve and return in terms of uh, speech and language listening skills again it can be negatively impacted” (PHC, 2)

**Impact of IED on motor development and physical activity**

“Depending on the children, it was things like they could you use iPad? They could get on to any site they wanted. But it was basic things like they couldn't walk up the stairs. So, it's as though it, you know. So, they were spending that much time on the iPads or phones or whatever that they were missing out on those basic essential skills like balance, coordination, and spatial awareness, and I think that spatial awareness might go back to that jigsaw thing where they didn't have that spatial awareness. When they were playing, you know, in terms of spatial awareness, they didn't have any, you know? It was shocking, really, because like, at that age now, they should be able to be able to go out and run around and not walk or bump into each other like they are doing. You know, they just had no concept of that wider vision of what's going on wider. You know, so yeah, so that was surprising” (PHC, 1).

“Again, that is because they're not going out and playing in that garden or whatever or being allowed to take those risks like balancing and doing stuff that children should be doing to learn all these skills and then again, where does that lead them in future?” (PHC,1).

“If you've got children who are spending a long amount of time on interactive media, they may not be getting that physical activity that they need, so they may not be out doing the climbing…” (PHC, 2).

**IED use in nursery settings**

“I found that teachers relied a lot on iPads and interactive technology to deliver lessons. So, in terms of going to YouTube. I found that from early years, upward things, like the role of science and things, rather than doing it hands-on. It was easier, and this is just my experience. The teachers seem to find it easier to do it online because it is less hassle than having to set up resources and tools to get the children all sorted out to do that practical lesson. What came from that is that the children missed out on that experience or things like you know, like if you're doing science, you're doing around flowers or whatever. Things like that, so is that smell, that touch, that interaction, that interaction with nature. All those things that fine motor skills are having to, you know like use tools, use resources. So, I found that a lot of children missed out on the on, on those experiences because the teachers relied so much on being able to go into the interactive whiteboards and use those and also with iPads. So, a lot of the children brought in their iPads, so a lot of work was done on the iPads.” (PHC, 2)

“They could do this on the iPad, but if you gave them an actual jigsaw, that brain connection just didn't figure out they didn't know how to put a jigsaw together. But we could do it on the iPad by swiping the finger, so you could so they didn't have that fine motor skills on that like conception to be able to put those pieces of that jigsaw together in real life, but they could talk about doing it on an iPad absolutely fine on an iPad, so again we had a bit of concern about what was going on there when they could do on an iPad. But actually, when you gave them for Jigsaw, they were unable to put those pieces together. Erm, because they couldn't physically do it, you know, they have those fine motor skills and like a say. It just seemed as though something was missing with that connection of to do it.” (PHC,2)

“So, all those essential skills that were needed were being missed because the teachers went on to use the iPads rather than that practical session. And I think it's probably down to time and there's we found a lot of pressure from managers who weren't early years trained. Because they wanted those students to meet those objectives. And it wasn't, and it was a quick lesson, and you moved on quickly rather than it being a flexible lesson. So, because of time constraints, I think they found it just easier to go on the iPads, get it done, dusted, and get the lessons finished. But I personally felt that the children were missing out greatly on loads of experience and learning development opportunities that they could have had” (PHC, 1).

“The other side of it, though, is when I was the outdoor learning sort of teacher. If you like, all my classroom outdoors is role play based; we're all done round. Even like the reception children, we're all done round play, but now and again, it was really good to have access to an iPad. So, things like if you were doing about sort of like, Antarctica or whatever, you could show what it looked like, and this was in Dubai. You could show. Things like that. So if it was, you know you, you talked about Antarctica. The children didn’t have any visual knowledge of what Antarctica looked like. So, there were those opportunities where we can actually look at this. This is what Antarctica looked like, or if you're doing things around it like bugs or insects or whatever, those are here. Well, actually, let's have a look at the Internet and see what those look like let's find out.” (PHC,2)

“And I think some of the drawbacks are, is that some of the adults working with young children are maybe not as familiar with the technology as the children, and perhaps see it as a negative. It does have its negatives. Research has shown some of that. There are concerns about what they can access. What's available, and the fact that it's available 24/7? On the other hand, there are a lot of positives to it. It is a world that they'll grow up in. It's a world that they'll work in, so they need their skills to be able to do that” (PHC,2).

**Intervention development**

“It has to be started in the early years because the children are growing up in a digital age, so they are using interactive technology, and you know, as they become adults, that will be their world. You know, a lot of people now don't write, they don't handwrite, everything's typed. We used a screen as part of everyday life, and we were already getting there before we went into COVID, and I think it's like learning. It's like teaching children to cross the road. We teach them to cross the road safely. We teach them to use roads, pavements, et cetera. In a safe way, that's part of learning, and I think interactive technology needs to be seen in the same way.” (PHC1)

“I definitely think that needs to be some, you know, like guidance and things and look at, you know, like and not just sort of like the guidance but explain why. Because the children and the parents need to know why. Well, actually, they don't need iPads. They need to play. They need to go out. They need to meet friends. They need to make mistakes. They need to climb trees. They need to fall down. They need to hurt themselves. They need to take risks. They need to develop different emotions. Things like fear. You know if you're like you because you know it happens during your life. And if you haven't got those emotions or those things in place. You're going to have problems later, you know. And I always used to say to parents. It's great that your child is really academic, and they can leave school with like, I don't know 10 GCE's or whatever, but if they can't communicate with other people or they can't socialise and they haven't got those skills to be able to get on the bus by themselves, for example. You know you're going to have problems” (PHC, 2)

“What I'd like you to do is get a bit of feedback on whether that worked. Because when we do training with our settings, what we do is give it a couple of weeks, and we will ring up and say, right, what is the impact? Did you do it? You know, has it worked out? What did you get from it? Was it a way of time? So, it’s on antidote stuff, dead quick stuff, and it could happen with the parents, so you could put this on and say, can we contact you if, you know, 3 - 4 weeks’ time, or whatever, just to see if it's changed your mindset? Of you know, what you’re doing with your children sort of thing?” (PHC, 1)

“So, it has to be consistent messages across all. So, what are children learning that our parents are being given? What do the settings know, and how do we build on those different stages? How do we keep up to date with technology as well? I think one of the things in this particular field” (PHC,2).

“As technology develops as things change, they need to feel that they are confident to keep up to date and abreast of that. I also think that there would need to be a little bit about how they speak to parents about what they do in their settings, particularly if they've got parents who are very anti-using the technology” (PHC, 2).
